# Optimized Slurries for Spray Drying: Different Approaches to Obtain Homogeneous and Deformable Alumina-Zirconia Granules

**DOI:** 10.3390/ma6115382

**Published:** 2013-11-21

**Authors:** Valentina Naglieri, Dan Gutknecht, Vincent Garnier, Paola Palmero, Jérôme Chevalier, Laura Montanaro

**Affiliations:** 1 Department of Applied Science and Technology, INSTM R.U. PoliTO, LINCE Lab., Politecnico di Torino, Corso Duca degli Abruzzi, 24, 10129 Torino, Italy; E-Mails: vnaglieri@lbl.gov (V.N.); laura.montanaro@polito.it (L.M.); 2 Now at Materials Science Division, Lawrence Berkeley National Laboratory, Berkeley, CA 94720, USA; 3 Université de Lyon, INSA de Lyon, MATEIS UMR CNRS 5510, Bât. Blaise Pascal 7, Av. Jean Capelle, 69621 Villeurbanne, France; E-Mails: dan.gutknecht@yahoo.fr (D.G.); vincent.garnier@insa-lyon.fr (V.G.); jerome.chevalier@insa-lyon.fr (J.C.); 4 Institut Universitaire de France, 103 bd Saint-Michel, 75005 Paris, France

**Keywords:** Al_2_O_3_-ZrO_2_, slurry optimization, spray drying, granules morphology

## Abstract

Spray drying is widely used for producing granulated feed materials for compaction process, which is the current industrial method for manufacturing alumina-zirconia femoral heads. The optimization of the granules compaction behavior requires the control of the slurry rheology. Moreover, for a dual-phase ceramic suspension, the even phase distribution has to be kept through the atomization step. Here we present two approaches addressing the key issues involved in the atomization of a composite system. Alumina-10 vol % zirconia powders were prepared by either a powder mixing route, or by the surface modification of a commercial α-alumina powder with a zirconium salt. Slurries from both powders were spray dried. The correlation between slurry rheology and pH, granules morphology and sintered microstructures was here investigated and discussed on the ground of the two feed materials characteristics. The processing conditions were optimized to obtain dense and homogeneous alumina-zirconia micro-nano composites by both processing routes.

## 1. Introduction

Zirconia-Toughened Alumina (ZTA) composites have raised great interest in the orthopaedic field [1], being particularly suitable for the production of femoral heads due to their biocompatibility and excellent room-temperature mechanical properties such as strength, toughness and wear resistance [2,3,4]. However, the performance of ZTA composites is deeply affected by their microstructural features [5,6,7,8]. Indeed, a good dispersion of zirconia in the alumina matrix is a key issue to produce reliable biomedical devices with the required mechanical behavior and free from low temperature degradation (LTD), as zirconia aggregates can lead to localized aging phenomena [8,9,10]. In addition, to fully exert their transformation toughening effect, the tetragonal zirconia grains should range between two critical dimensions: *d_c_*’, below which the tetragonal-to-monoclinic transformation is hindered, being the tetragonal zirconia stabilized by its small dimensions; and *d_c_* above which spontaneous transformation occurs during cooling from the sintering temperature [11]. In this frame, the production of homogeneous alumina-zirconia composite powders, with controlled microstructural features, is a key requirement for achieving the expected performance.

The current industrial production of ceramic femoral heads implies shaping by dry pressing of granulated powders. In particular, femoral heads manufacturing usually involves a few basic steps: powder atomization, cold isostatic pressing, sintering, post hot isostatic pressing (post-HIP) and a final step of machining and polishing [12]. Thus, even if a highly homogeneous composite powder is provided, heterogeneities could be introduced during the following manufacturing steps. Although compaction offers some major advantages such as rapid shaping, automation and no product-drying requirement, it is prone to internal defects that can limit the strength of the final parts [13]. Hence, the characteristics of the granules have to be optimized to improve the compaction behavior and reduce the number and size of the flaws. The relationship between the granules characteristics and the slurry rheology has been widely investigated in literature [13,14,15,16,17,18,19,20].Indeed, the shape, density, strength and deformability of the granules depend on the interparticle forces and, thus, on the slurry viscosity. Particularly, a flocculated slurry can produce low-density solid granules, easy to be deformed and pressed, where as a deflocculated suspension can yield hollow and dense granules, which are hard to be pressed and can give rise to large pores in the sintered ceramics [14,17,20].

Another source of flaws that can be found in ceramic parts obtained by compaction of atomized powders is the incomplete cohesion of granules, which can lead to crack-like defects at the granules boundaries. This behavior has been imputed to an uneven distribution of the binder in the pressed bodies, caused by its migration toward the granule surface during drying [21,22,23]. Thus, also the binder plays a major role on the granules compaction behavior affecting the mechanical properties of the final components.

Aim of this work is to investigate the main critical issues concerning the spray drying of alumina-zirconia composites slurries, with the purpose of achieving flaw-free and highly homogeneous sintered composites. In fact, in spite of the extensive literature on the atomization of a single-phase powder, only few works relate to spray drying of composite systems, such as alumina-zirconia [24,25]. The atomization of a dual-phase ceramic suspension involves some additional problems compared to the single-phase ones. Indeed, alumina and zirconia powders must be simultaneously dispersed, in spite of their different dispersion behavior and conditions [8], to assure a good phase distribution in the composite granules. Otherwise, a successful atomization requires the use of flocculated suspensions [14], being this condition quite in conflict with the previous one.

In a previous paper [26], ZTA composite materials containing respectively 5, 10, 15 and 20 vol % of un-stabilized zirconia were prepared by slip casting and pressureless sintering. A preliminary mechanical characterization showed that composites containing 10 vol % ZrO_2_ exhibited good hardness and the maximum fracture threshold. Based on those results, alumina-10 vol % ZrO_2_ (AZ) powders prepared by using two different methods are here presented. In the first case, the composite slurry was produced by powder mixing of commercially available raw oxides; in the second one, a commercial α-alumina powder was submitted to a surface modification process [27,28,29] by using an aqueous solution of a zirconium salt. In this latter process, nano-crystalline tetragonal zirconia grains directly crystallized on the alumina particles surface under controlled thermal treatments [30] forming a composite powder used to prepare the slurries to spray dry. A correlation between slurry rheology, granules morphology and sintered microstructures is investigated and discussed, focusing on the characteristics of the two feed materials, namely the granules obtained by powder mixing (PM) of commercial powders, and those obtained from the composite powder, which was prepared by the surface modification (SM) route. According to these two preparation methods, powders derived by the powder mixing and surface modification routes are hereafter referred to as PM-AZ and SM-AZ, respectively.

## 2. Results

### 2.1. Powder Mixing Procedure

**Figure 1:**
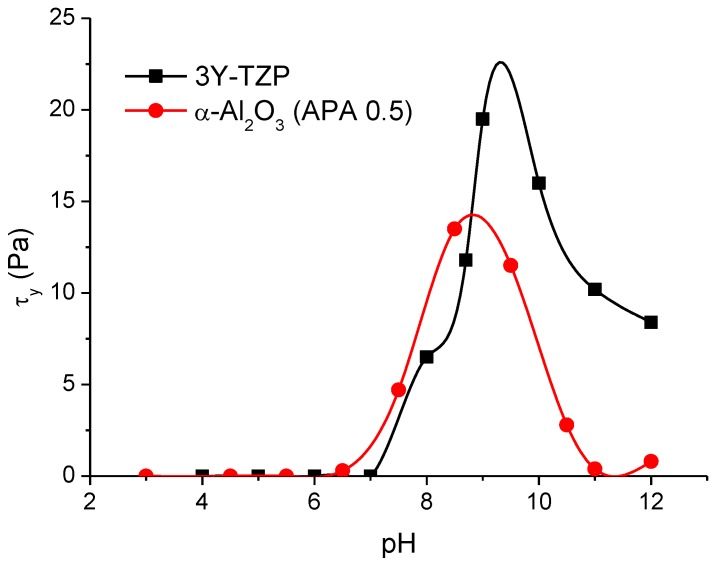
Yield stress (τ*_y_*) as a function of pH for α-alumina (APA-0.5) and 3Y-TZP suspensions used in the PM procedure.

The role of the pH on the suspension rheological behavior was first evaluated on pure α-alumina and yttria (3 mol %) stabilized zirconia (here after referred to as 3Y-TZP) powders. The shear stress versus shear rate curves were recorded at different pH values and fitted by Casson equation [31]. Figure 1 shows the yield stress (τ*_y_*) as a function of pH for both reference materials. As the flocculation conditions correspond to high yield stress values, the curves depicted in the Figure 1 allow to define the flocculation pH range for both powders. Precisely, α-alumina shows a well-defined flocculation range, between pH 6.5 and 11, whereas 3Y-TZP shows a very low yield stress up to pH 7, followed by an increase of τ*_y_* while increasing the pH. From these results, PM-AZ suspension was first dispersed at pH 4.5, as this condition denotes a well deflocculated state for both alumina and zirconia powders. Then, the pH was progressively increased, inducing electrostatic destabilization, which caused the yield stress to increase.

**Figure 2:**
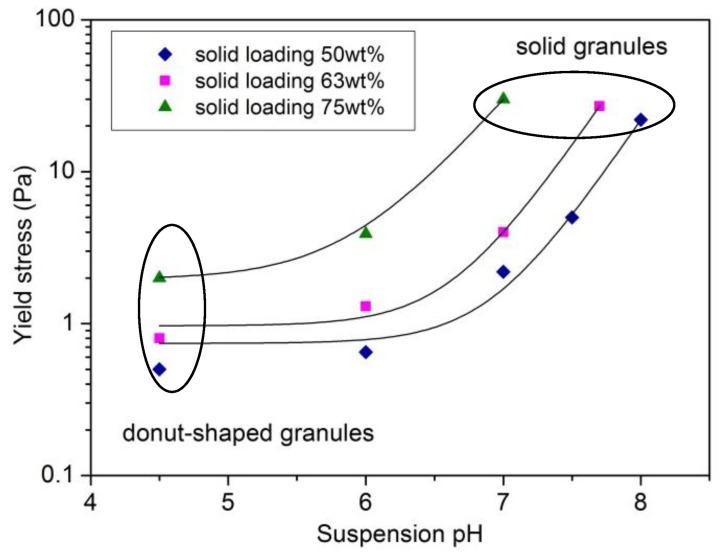
Yield stress as a function of pH for PM-AZ suspensions.

Figure 2 depicts the τ*_y_* values as a function of pH for PM-AZ suspensions at different solid contents. The yield stress increases by increasing the suspension pH and the solid loadings. However, it was observed that at the higher solid contents (such as 63 and 75 wt %), the electrostatic destabilization produced the formation of macroscopic flocs, whose presence could induce microstructural inhomogeneity.

**Figure 3:**
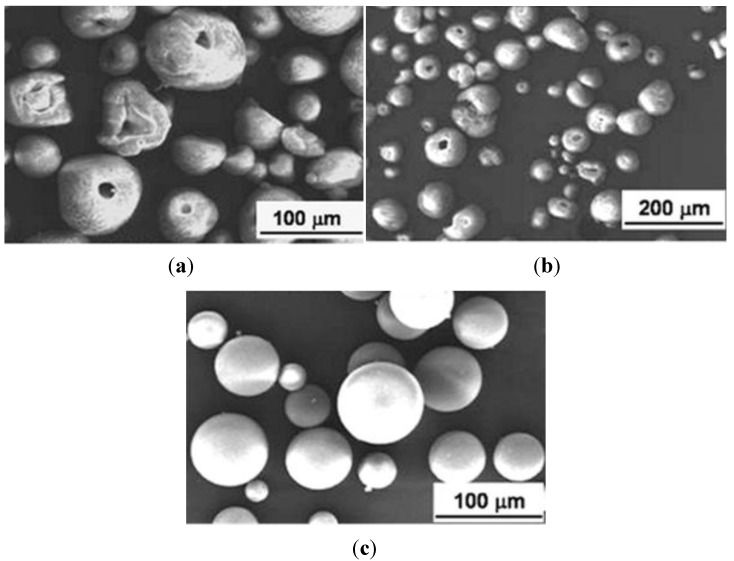
PM-AZ granules obtained by spray drying a (**a**) deflocculated suspension at pH 4.5; or destabilized suspensions at (**b**) pH 7; and (**c**) at pH 8.

As a consequence, only suspensions with 50 wt % solid content were selected for the subsequent atomization step. It is known [13], however, that the rheological features of the suspensions affect the morphology of the spray dried granules; as a general trend, deflocculated suspensions (characterized by a low yield stress) yields donut-shaped or hollow granules, whereas properly flocculated suspensions lead to solid granules, without a central cavity. The terms here used to describe the granule morphology agree with denominations previously proposed in literature [18]. As a consequence, PM-AZ granules obtained by the deflocculated suspension at pH 4.5 and after electrostatic destabilization at pH 7 and 8 were submitted to a microstructural characterization (Figure 3a–c). We can observe that when the ceramic slurries had low yield stress (of about 0.5 and 2.2 Pa, at pH 4.5 and 7, respectively) the granules showed a large, central void; whereas slurries with τ*_y_* of > 20 Pa yielded solid granules.

The tap densities of granules were the following: 32.2% of the theoretical density (TD) when a deflocculated suspension was used; 29.8% TD and 28.6% TD when suspensions destabilized at pH 7 and 8, respectively, were spray dried. However, in the first two cases the granules presented a large, central void (see Figure 3); thus, it was assumed that the density of their shell was higher than the measured mean value. On the opposite, a lower density characterized the solid granules.

**Figure 4:**
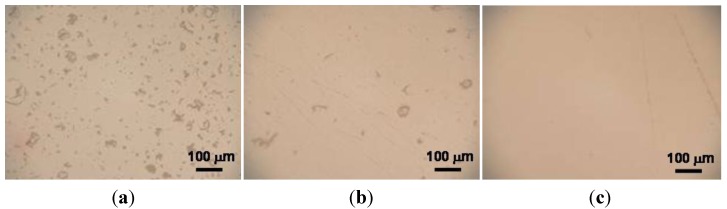
Polished surfaces of PM-AZ sintered samples obtained by granules from deflocculated suspension at (**a**) pH 4.5; from destabilized suspension at (**b**) pH 7; and (**c**) pH 8.

**Figure 5:**
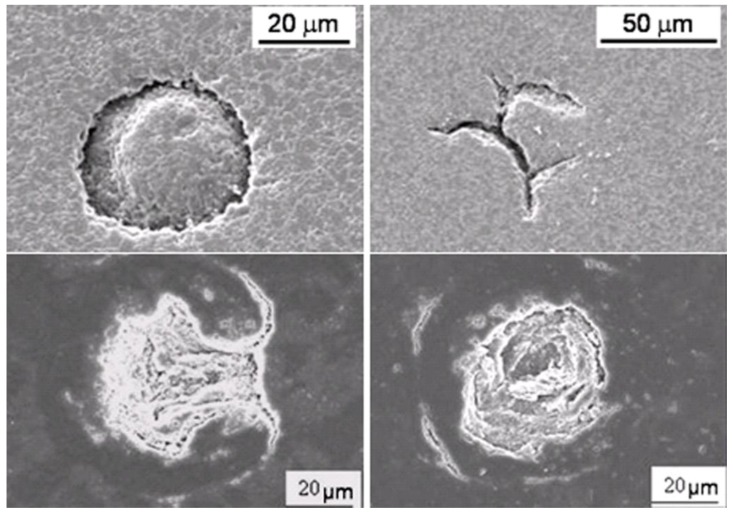
Examples of typical defects observed on the surfaces of PM-AZ samples, obtained by pressing hollow granules.

**Figure 6:**
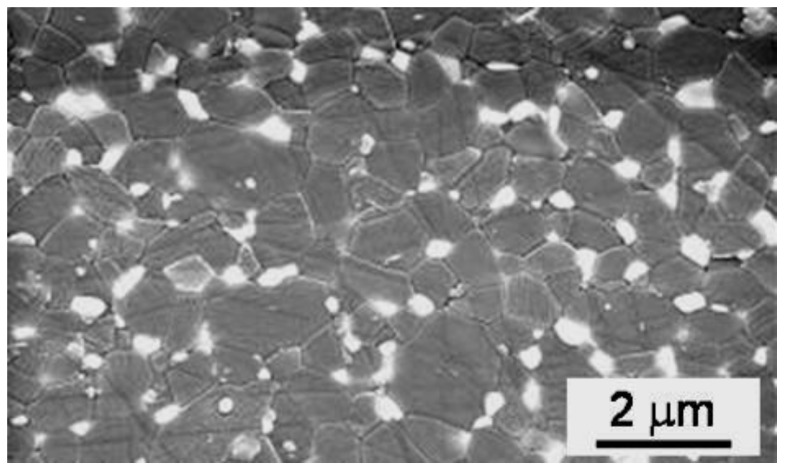
Environmental scanning electron microscope (ESEM) image of the microstructure of the sintered PM-AZ composite obtained from optimized, solid granules.

Granules, obtained by the three above suspensions, were isostatically pressed, giving rise to green pellets having a density of about 56.5% TD, regardless the granule morphology. After pressureless sintering at 1520 °C, these samples achieved a density >97% TD; finally, after post-HIP they reached full densification (≥ 99.9% TD). Polished surfaces were observed by optical microscopy (Figure 4), showing the presence of several, large defects in the materials obtained from hollow granules (Figure 4a,b), whereas solid granules yielded defect-free samples (Figure 4c). The SEM micrographs in Figure 5 give more clear details about the aforementioned defects, produced by pressing hollow or non-deformable granules. Figure 6 shows a higher-magnification image of PM-AZ produced from solid granules, demonstrating that a highly homogeneous microstructure was successfully obtained by performing a controlled electrostatic destabilization.

### 2.2. Surface-Modification Procedure

**Figure 7:**
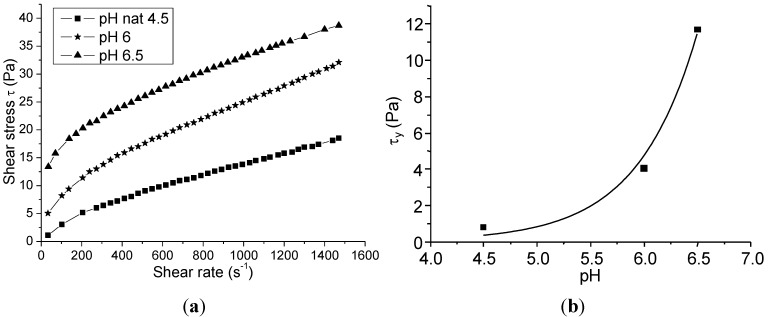
(**a**) Shear stress vs. shear rate for SM-AZ suspensions at different pH values; and (**b**) yield stress of the SM-AZ suspension as a function of pH.

The rheology of SM-AZ suspension was evaluated at pH 4.5 (natural pH), 6 and 6.5. Higher pH values were not considered, leading to very high suspension viscosity. The shear stress *vs.* shear rate curves for these suspensions are shown in Figure 7a. An increase in the pH induced higher shear stress values. These data were fitted by the Casson equation, in order to evaluate the yield stress, as plotted in Figure 7b. The increase of yield stress by increasing the pH denotes more flocculated slurries.

**Figure 8:**
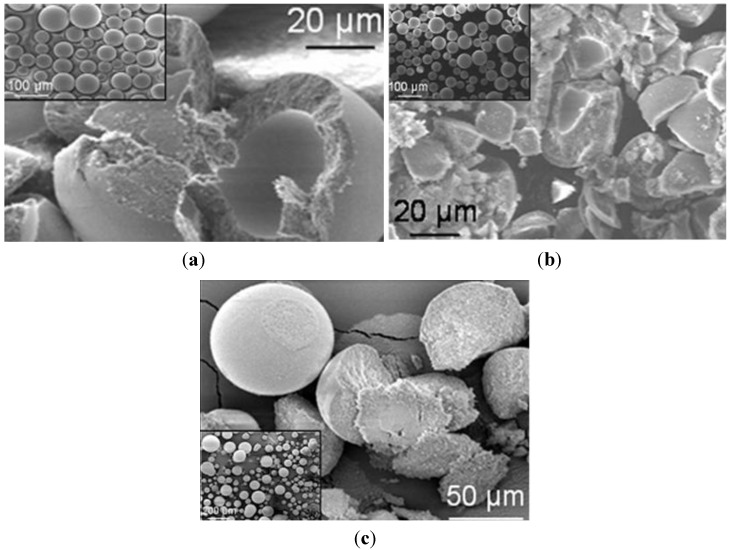
SEM images of granules obtained by spray drying SM-AZ suspensions at (**a**) pH 4.5; (**b**) pH 6; and (**c**) pH 6.5 (a low magnification image in the insert).

The three suspensions were then spray dried. All the SM-AZ granules (Figure 8) were spherical, showing almost smooth and defect-free surfaces, independently from the starting suspension pH. However, microstructural observations performed on intentionally crushed granules allowed to highlight significant differences due to the yield stress of the starting suspensions. In fact, slurries characterized by low yield stress gave rise to hollow granules, whereas the granules walls thickened as the yield stress increased. Finally, when suspensions properly flocculated were used, full or solid granules without central voids were successfully obtained. The tap densities were: 32%, 33% and 28% TD, for the powders obtained from suspension at pH values of 4.5, 6 and 6.5, respectively. Thus, the mean granule density decreased in the case of the better flocculated suspensions.

**Figure 9:**
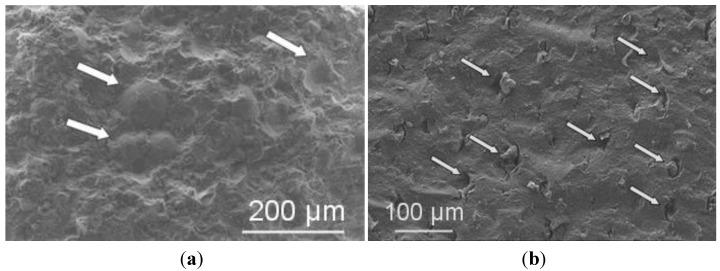
Fracture surfaces of (**a**) green; and (**b**) sintered bodies obtained by isostatically pressing SM-AZ hollow granules.

The atomized granules were then isostatically pressed, reaching a green density of about 57% TD, regardless the suspension pH, and then pressureless sintered, reaching a final density of about 97% TD. In Figure 9, SEM micrographs of the fracture surfaces of green (Figure 9a) and sintered (Figure 9b) samples obtained by pressing hollow granules, are shown. Several defects can be easily observed, imputable to the use of not-optimized granules.

**Figure 10:**
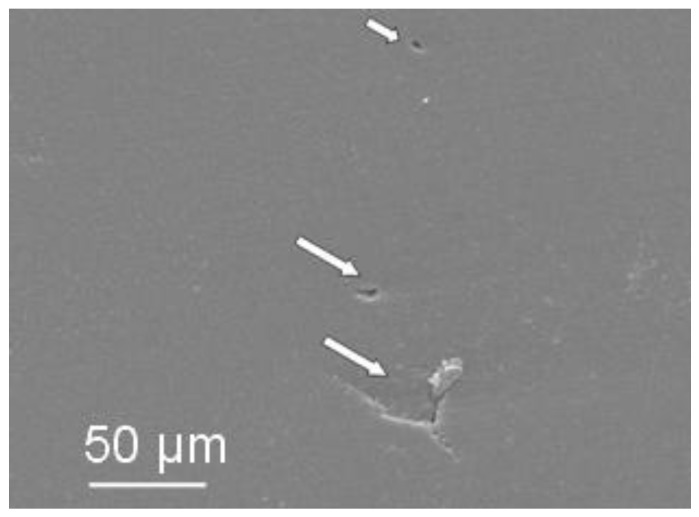
Fracture surface of a sintered sample obtained by isostatically pressing SM-AZ solid granules.

**Figure 11:**
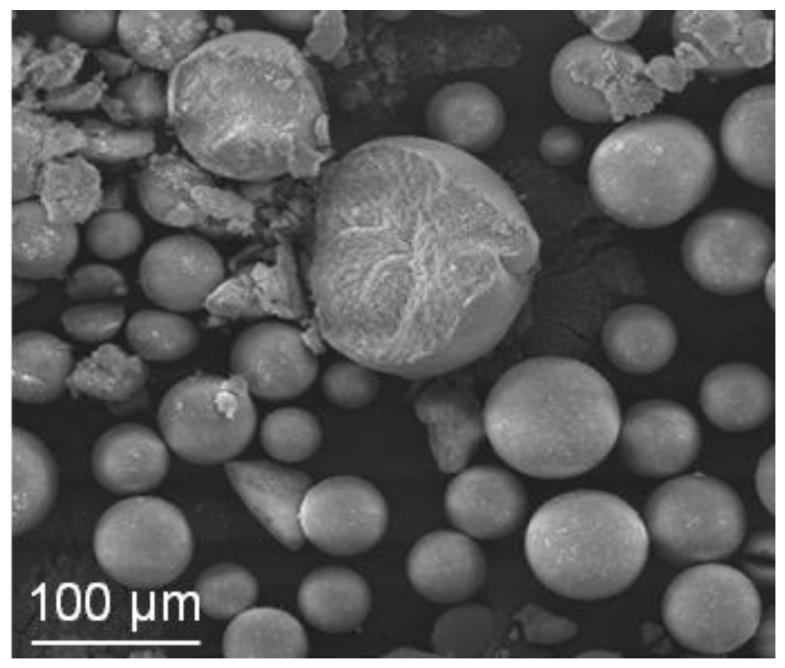
SEM images of granules obtained by spray drying SM-AZ suspensions destabilized at pH 6.5, with 3 wt % PEG 4000.

**Figure 12:**
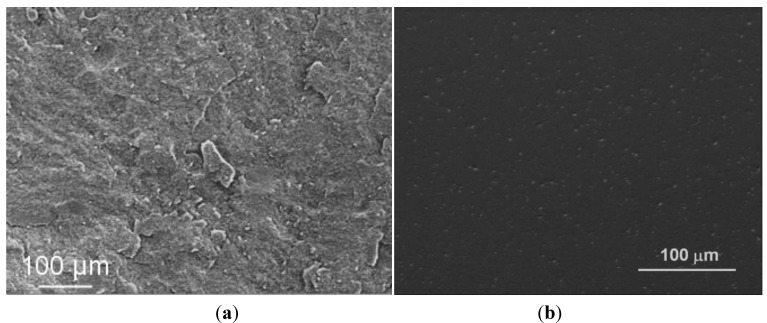
(**a**) Fracture surface of a green compact; and (**b**) polished surface of a sintered sample obtained by SM-AZ granules containing 3 wt % PEG 4000.

**Figure 13:**
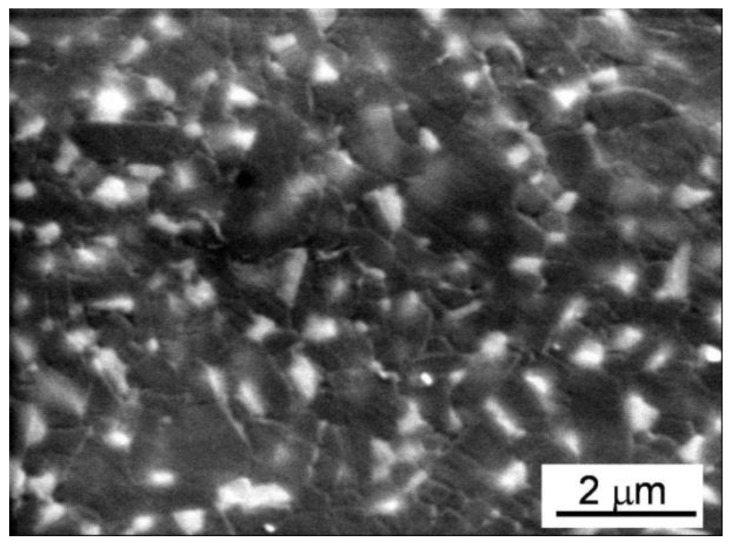
SEM micrograph of the sintered SM-AZ composite obtained by dry pressing the optimized granules.

Although a significant improvement in the sintered microstructure homogeneity was obtained when solid granules were processed, some large intergranular defects were still present (see Figure 10). Thus, for increasing the reliability of these ceramics and to match the standard for biomedical applications, the role of the binder amount was investigated. A SM-AZ suspension, destabilized at pH 6.5, was added with 3 wt % PEG 4000 and then spray dried. Once again, the granules were spherical, full and defect-free, as shown in Figure 11, with a tap density of 29% TD, similarly to SM-AZ processed at pH 6.5 with 1 wt % binder. However, in this case both green compacts and sintered bodies were homogeneous and defect-free, as shown in Figure 12. The small defects on the sintered surface of Figure 12b are due to the pull-out of some fine zirconia grains during polishing. Finally, Figure 13 shows a higher-magnification image of SM-AZ composite, processed in the proper conditions (*i.e.*, at pH 6.5 and with 3 wt % binder), showing a very homogeneous microstructure in which zirconia grains are well distributed into the alumina matrix.

## 3. Discussion

In this study, the rheological characterization of the ceramic suspensions was exploited as a powerful tool for optimizing the spray drying process. Particularly, Walker *et al.* [13] highlighted the influence of the suspension rheological characteristics, mainly expressed by the yield stress *(*τ*_y_*) and the limit viscosity at infinite shear rate (η_∞_), on the granules features. As already stated [13], τ*_y_* and η_∞_ have a clear physical significance for a flocculated slurry, being related to the interparticle forces. Interparticle attractive forces allow the formation of a network of weakly bonded particles, giving rise to a local, continuous structure of the slurry in the form of flocs. In this condition, τ*_y_* denotes the yield stress required to initiate flow. As the slurry is sheared, the floc structure breaks down, and at high shear rates the viscosity approaches η_∞_. Here, for both PM-AZ and SM-AZ systems, the degree of flocculation increases by increasing the suspension pH toward their IEP, thus providing a higher τ*_y_*. In addition, we observed that low-yield stress suspensions led to hollow granules, while the slurry whose τ*_y_* was >20 Pa for PM-AZ and >11 Pa for SM-AZ provided the suitable rheological conditions to obtain solid granules. These results agree with the model proposed by Walker *et al.* [13], and then confirmed by other Authors [17,18,19], which describes the granule formation during spray drying. In fact, according to the Walker’s model, the granule density and shape depend on the drying mechanism through which the suspension drops transform into dry granules. The degree to which particles are able to rearrange during drying is determined by the strength of the floc structure, as indicated by the slurry yield stress. In low yield stress suspensions, the ceramic particles can easily move and migrate toward the granule surface as water is removed. This gives rise to a large, inner void and to a dense, outer shell. Otherwise, when the slurry yield stress is sufficiently high, the particles can hardly move and cannot be drained during the liquid evaporation because of the insufficient shear stress. Thus, the outer shell does not form and solid granules, without the central void, are produced [13].

In our case, the critical yield stress to obtain solid granules was about 11 and 20 Pa for PM-AZ and SM-AZ, respectively. Moreover, we have shown that for intermediate τ*_y_* values (of about 2 Pa for PM-AZ and 4 Pa for SM-AZ), the granules showed hollow shape but with an increased shell thickness with respect to the case of deflocculated slurries. Thus, a quantitative correlation between the suspension rheology and the granule morphology is given by means of viscosimetric measurements and related calculation of the yield stress.

PM-AZ and SM-AZ atomized powders also showed a different pressing behaviour as a function of the rheology of the starting ceramic slurry. In fact, pressed samples produced from deflocculated suspensions present several large flaws, due to the poor deformability of the granules, which in turn was related to their density. In fact, in deflocculated suspensions, the particles were well packed and gave rise to high-dense granules [13], difficult to be pressed. As a result, the large internal cavities of hollow granules or/and the intergranular pores often persist after pressing and sintering, as shown in Figure 9. In the case of PM-AZ pressed samples, the flaws completely disappeared when granules were produced by flocculated suspensions. On the opposites, SM-AZ specimens still exhibited defects mainly imputable to a memory of the interfaces between granules. Such discrepancy in pressing behavior cannot be explained on the ground of the tap density, being similar for SM-AZ and PM-AZ. Thus, it was assumed that the SM-AZ granules were harder than the respective PM-AZ ones. The compressive strength of a single granule is influenced by the coordination number and adhesive forces between the primary particles, as stated by Naito *et al.* [14], who observed that the compressive strength of Si_3_N_4_granules could be different even if they exhibited the same porosity. Supposing that the coordination number of the primary particles depends on the porosity, they concluded that the adhesive forces were affected by the slurry preparation, and particularly by the dispersant concentration [14]. On the basis of this hypothesis, the different deformability of PM-AZ and SM-AZ granules can be explained, considering the smaller particle size and the higher specific surface areas of the SM powder, as compared to the PM one (see Table 1). According to such physical features, the attractive force between primary particles of the SM-AZ can be reasonably assumed to be higher than that of PM-AZ. SM-AZ granules with low binder content (1 wt %) did not show suitable deformability, whereas, when the binder amount was increased to 3 wt %, defect-free samples were successfully produced. This result strengthens the aforementioned hypothesis, highlighting the role of powders characteristics (specific surface area and particle size) on the ceramic processing parameters.

## 4. Experimental Section 

### 4.1. Commercial Powders

Commercial alumina and zirconia powders were selected in order to meet ISO 6474 and ISO 13356 specifications for the biomedical grade ceramics. For the powder mixing (PM) route, sub-micrometer α-alumina (Ceralox APA 05, Hamburg, Germany) and yttria-stabilised zirconia (Tosoh Corporation, Tokyo, Japan) powders were used. For the surface-modification (SM) process, nanocrystalline alumina powder (Taimei Chemical Co., Tokyo, Japan) was employed. The main powder features are collected in Table 1.

**Table 1:** Main features of the commercial powders.

Powders	Mean particle size (μm)	BET specific surface area (m^2^/g)	Impurities (%)	Isoelectric point (pH)
α-Al_2_O_3_ APA-0.5 (Ceralox APA 05, Hamburg, Germany)	0.35	8.1	0.04	9.5 [8,32]
ZrO_2_ (3Y-TZP, Tosoh Corporation, Tokyo, Japan)	0.6	14	≤ 0.07	10 [8,32]
α-Al_2_O_3_ TM-DAR (Taimei Chemicals Co., Tokyo, Japan)	0.10	14.5	≤ 0.01	9 [33,34]

### 4.2. Powder Mixing Technique

Suitable amounts of alumina and 3Y-TZP powders were mixed to prepare Al_2_O_3_-10 vol % ZrO_2_ (referred to as PM-AZ). Aqueous suspensions at three different solid loadings (50, 63, and 75 wt %) were prepared and dispersed by ball milling, performed using high purity alumina balls in a plastic jar for 24 h. According to the isoelectric point of the two raw materials, which is displayed at basic pH values (see Table 1), the suspensions pH was adjusted to 4.5 in order to induce a simultaneous electrostatic dispersion of the two powders [8].

### 4.3. Surface Modification Technique

The surface modification route of α-alumina nano-powders was carried out to prepare Al_2_O_3_-10 vol % ZrO_2_ composites (referred to as SM-AZ). The technique consists on the addition of a suitable amount of zirconium chloride aqueous solution to a well dispersed alumina suspension. As for PM-AZ, dispersion of the alumina powders was carried out in ball-milling, performed with high purity alumina balls in a plastic jar for about 12 h, at a pH value of about 3.5. The obtained slurry, made of a mixture of alumina particles and metal salts, was spray dried, and then calcined at 600 °C for 1 h, under air, to promote by-products burn-out and to induce the crystallization of zirconia nano-grains on the surface of the α-alumina particles [30]. In this process, any zirconia phase stabilizer was added, since the tetragonal phase was retained at room temperature due to the ultra-fine size of the zirconia particles. In fact, as given in previous work, after this thermal treatment, tetragonal ZrO_2_ particles of about 10–20 nm were observed by TEM [30]. The specific surface area of the calcined powder was 15 ± 0.5 m^2^/g [35], slightly higher than the starting nano α-alumina one. Powder suspensions of the doped alumina with a solid loading of 63 wt % were dispersed by ball-milling at the natural pH value of about 4.5.

### 4.4. Viscosity Measurements

Pure α-alumina (APA 0.5) and 3Y-TZP powders, here used as a reference, were first separately dispersed by ball-milling at 35 wt % solid loading. The slurries pH was slowly modified from its natural value by adding dilute NH4OH or HCl, and the evolution of the rheological behaviour was evaluated by using a Couette Rheometer (HAAKE VT 501, Villebon, France). The shear stress (τ) was measured as a function of the shear rate $\dot{\gamma }$ in the range 0–1500 s^−1^. The obtained curves were fitted by using the Casson equation [31]:

τ^1/2^ = τ*y*^1/2^ + η_∞_^1/2^*γ̇*^1/2^ (1)

where η_∞_ is the viscosity limit for infinite shear rate and τ is the yield stress, which is an index of the flocculation degree, being an indirect measure of the interparticles forces [13].

PM and SM composite powders were dispersed by ball milling at a pH value of about 4.5. Then, the pH was slowly changed to induce flocculation and viscosity measurements were performed at each pH values.

### 4.5. Spray Drying

1 wt % PEG 4000 binder [15] was added to PM and SM suspensions, at different pH values. The binder addition did not modify the rheological behaviour of the suspensions, as verified by viscosimetric analyses. Then, the ceramic slurries were spray dried using an ultrasonic spray dryer (Sodeva ATSELAB, Chambery, France). The frequency of the ultrasonic probe was 20 kHz, the inlet and the outlet temperature were 180 °C and about 85 °C, respectively. Several spray drying runs were performed, using different suspensions, to determine the influence of the suspension flocculation degree, if any, on the granule morphology. The granule morphology was observed by SEM (Hitachi S2300, Tokyo, Japan) and ESEM (FEI, ESEM XL30, Hillsboro, OR, USA). The tap density was determined in accordance with the standard NF ISO 3953, and compared to the theoretical density of α-Al_2_O_3_ 10 vol % tetragonal ZrO_2_ composite (4.17 g∙cm^−3^).

### 4.6. Forming and Sintering

Granules were first uniaxially pressed at 150 MPa and then isostatically pressed at 350 MPa, giving rise to small pellets of 10 mm in diameter and 3 mm in thickness. These samples were pressureless-sintered at 1520 °C for 1 h (PM–AZ) [32] or at 1500 °C for 3 h (SM–AZ) [26] and then submitted to post-HIP at 1520 °C for 1h, under a pressure of 200 MPa [32]. The green microstructures were observed by ESEM. The sintered samples were observed by optical (Zeiss Axiophot, Jena, Germany) and electron microscopy (ESEM and SEM). The density of green and sintered samples was evaluated from mass and geometrical measurements and by the Archimedes method.

## 5. Conclusions

In this study, we have shown two successful approaches to address the critical issues related to the production of ZTA composites by spray drying and pressing. Indeed, an even phase distribution and a defect-free microstructure are paramount requirements for composite parts such as ZTA femoral heads. The first proposed method comprised a powder mixing technique. A key point of this procedure was the control of the dispersion conditions, in order to avoid differential powder aggregation. In the second approach, a composite powder obtained by a surface-modification technique was used, in which tetragonal zirconia nano-grains were yielded onto alumina particles surface. Both ceramic slurries were atomized, and the relationship between the slurry viscosity and the features of the obtained granules was investigated. In both cases, low-yield stress suspensions gave rise to hollow granules, characterized by a dense and hardshell, whose poor deformability caused macroscopic flaws in green and sintered materials. On the contrary, solid granules were obtained by high-yield stress, flocculated suspensions. This resulted in defect-free sintered ceramic in the case of the mixed powder, whereas some flaws were observed in the material derived by the surface-modification route. Presumably, harder granules were produced by the second route due to higher adhesive forces between finer particles. According to such hypothesis, the binder amount was thus adjusted to yield deformable and easy to press granules also for the latter system. Finally, highly dense and very homogeneous ZTA materials were successfully produced by both the described processing routes through a careful control and optimization of the process parameters.
